# Targeting Mechanics to Restore Right Ventricular Function: A New Frontier in Pulmonary Arterial Hypertension Therapy

**DOI:** 10.1093/function/zqaf029

**Published:** 2025-07-04

**Authors:** Taryn Wilson, Lian Tian

**Affiliations:** Strathclyde Institute of Pharmacy and Biomedical Sciences, University of Strathclyde, Glasgow, G4 0RE, UK; Strathclyde Institute of Pharmacy and Biomedical Sciences, University of Strathclyde, Glasgow, G4 0RE, UK

## A Perspectives on “Distinct Right Ventricular Performance in Response to Acute Colchicine Treatment in Healthy and Diseased States”

Pulmonary arterial hypertension (PAH) is a devastating condition driven by progressive pulmonary vascular remodeling and marked by persistently elevated pulmonary arterial pressure, leading to the thickening and enlargement of the right ventricle (RV). Over time, this can result in RV failure and ultimately death. Despite the availability of pulmonary vasodilatory and antiproliferative agents and improved disease management, the mortality rate among patients with PAH remains high. No therapies are currently approved to directly target the RV—the organ, not the lungs, which mainly determines the patients’ survival. As RV failure remains the leading cause of death in PAH, developing RV-specific interventions is increasingly urgent.^1^^,^^2^

Molecular and cellular mechanistic studies as well as preclinical and clinical studies have improved our understanding of RV physiology, function and pathomechanisms in PAH. Currently pharmacological, RNA- and cell-based therapies are being sought to directly modulate RV metabolism, inflammation, microvascular rarefaction, fibrosis, or biomechanics, etc., aiming to improve or restore RV function in PAH.^2^ It is worth noting that RV biomechanics and mechanobiology are being explored for potential therapeutic options.^3^^,^^4^ In PAH, the RV is not just passively subject to pressure overload but also undergoes structural and mechanical remodeling. The alterations in viscoelasticity, stiffness, and cytoskeletal architecture impair both diastolic and systolic function.^3^^,^^4^ The mechanical properties of the RV are increasingly recognized as both pathological and potentially modifiable.^4^

In this issue of *Function*, LeBar et al. present compelling evidence showing that targeting cytoskeletal mechanics via acute colchicine administration improves RV mechanics and function in male monocrotaline-induced PAH rats.^5^ This group has previously shown that acute colchicine treatment ex vivo reduced the passive viscoelasticity of RV from male monocrotaline-induced PAH rats.^6^ This study extended their previous ex vivo work by demonstrating that acute colchicine administration in vivo leads to immediate improvements in RV compliance, end-diastolic pressure-volume relation (EDPVR), preload, and stroke volume in male monocrotaline-induced PAH rats but no significant changes in RV (active) mechanics or function in healthy rats. Because PAH results in RV remodeling and increases RV viscoelasticity, partly due to the polymerization of microtubules, it is postulated that colchicine depolymerizes microtubules and reduces RV viscoelasticity, leading to improvement in RV mechanics and function.^5^

LeBar et al. employed pressure-volume measurements, enabling quantification of various systolic and diastolic parameters to fully characterize RV mechanics and function. The reduction in RV viscoelasticity evidenced by reduction in EDVPR and RV compliance in monocrotaline rats allowed for more efficient diastolic filling and enhanced contractile output evidenced by increased end-diastolic volume and stroke volume, respectively, leading to improved RV-pulmonary artery coupling and enhanced overall RV function. Importantly, these effects were observed only in the RVs exposed to chronic pressure overload. Healthy RVs showed no significant mechanical or functional change in response to colchicine, suggesting that the cytoskeletal alterations—namely, increased microtubule density—are specific to the pathological state. This specificity enhances the therapeutic appeal of cytoskeletal interventions: diseased tissue responds, while healthy tissue is spared.

This study reinforces an emerging paradigm in cardiovascular science: The RV is not merely a passive recipient of afterload, but a distinct biomechanical and immunological organ. Historically viewed as a low-pressure conduit, the RV is structurally, embryologically, and functionally distinct from its left-sided counterpart. Its thin wall, higher compliance, and sensitivity to afterload render it uniquely vulnerable in diseases such as PAH, where even modest increases in vascular resistance precipitate a cascade of maladaptive remodeling, reduced coronary perfusion, and ischemia.^3^^,^^7^ LeBar et al. demonstrate that this vulnerability may also be an opportunity. By targeting the microtubule network, their study shows that acute cytoskeletal modulation via colchicine leads to immediate gains in RV stroke work and compliance. This is not simply a hemodynamic shift, but a mechanical reprogramming: a reshaping of the internal forces that resist filling and contraction.^5^^,^^8^ This work is emblematic of a growing recognition that mechanics matter—not only as a reflection of pathology but also as a modifiable determinant of disease progression. Moreover, there is intriguing potential for synergy with current PAH therapies. While current PAH therapies work primarily by unloading the RV, colchicine may improve RV viscoelasticity and intrinsic contractility, opening the door to combination strategies.^1^^,^^9^

Moving forward, several challenges and opportunities will define the translational path. First, previous study has demonstrated the benefits of chronic colchicine treatment on RVs from male monocrotaline-induced PAH rats^8^; however, the effect of colchicine on RV in another widely used animal model, that is, the Sugen-hypoxia rat model, as well as the associated sex differences is unknown. Future research is needed to evaluate the effects of chronic colchicine treatment in both male and female Sugen-hypoxia rats. Second, clinical stratification tools that identify RV viscoelastic burden will be critical. Whether through imaging (eg, elastography, strain mapping), molecular markers, or tissue biopsies, the ability to noninvasively quantify myocardial stiffness and mechanoresponsiveness will help identify patients most likely to benefit. This could pave the way for a precision-medicine approach to RV failure. Mechanistically, this study by LeBar et al. opens doors to deeper exploration of how mechanical stimuli integrate with molecular signaling. For instance, mechanoresponsive transcription factors such as YAP/TAZ—shown to drive vascular remodeling and RUNX2, which interacts directly with TAZ to regulate osteogenic-like gene programs in pulmonary arteries, are key regulators of cardiac remodeling and fibrosis.^10^ Understanding how cytoskeletal modulation intersects with these pathways may yield synergistic or alternative targets with broader applicability across cardiac diseases. Finally, the concept of “viscoelastic reserve” may emerge as a clinically relevant construct. Just as afterload reduction has revolutionized RV heart failure management, reducing internal mechanical load in the RV may become a parallel strategy—one that restores contractile efficiency, prolongs adaptive remodeling, and improves outcomes in PAH and beyond.

In conclusion, by demonstrating the benefits of modulating RV mechanics on RV function in PAH, LeBar et al. move the field closer to a future in which biomechanics is not just a measured parameter but also modifiable and therapeutically targetable to treat cardiovascular diseases such as PAH.^5^
